# Gapless genome assembly and population genomics highlights diversity of mango germplasms

**DOI:** 10.1093/hr/uhaf007

**Published:** 2025-04-01

**Authors:** Cuixian Zhang, Huaifeng Yi, Xiuxu Ye, Jingxiao Fu, Dehong Xie, Tianqi Bai, Xinyue Gong, Zhangguang Ni, Xinping Luo, Yusuf Chong Yu Lok, Qiong Luo, Peng Wang

**Affiliations:** State Key Laboratory for Conservation and Utilization of Bio-Resources in Yunnan, Ministry of Education Key Laboratory of Agricultural Biodiversity for Plant Disease Management, College of Plant Protection, Yunnan Agricultural University, 452 Fengyuan Road, Kunming 650201, China; Institute of Tropical and Subtropical Cash Crops, Yunnan Academy of Agricultural Sciences, 518 Lancheng Road, Baoshan 678000, China; Institute of Tropical and Subtropical Cash Crops, Yunnan Academy of Agricultural Sciences, 518 Lancheng Road, Baoshan 678000, China; National Key Laboratory of Tropical Crop Breeding, Institute of Tropical Crop Genetic Resources, Chinese Academy of Tropical Agricultural Sciences, 4 Xueyuan Road, Haikou, Hainan 571101, China; National Key Laboratory of Tropical Crop Breeding, Institute of Tropical Crop Genetic Resources, Chinese Academy of Tropical Agricultural Sciences, 4 Xueyuan Road, Haikou, Hainan 571101, China; Institute of Tropical and Subtropical Cash Crops, Yunnan Academy of Agricultural Sciences, 518 Lancheng Road, Baoshan 678000, China; State Key Laboratory for Conservation and Utilization of Bio-Resources in Yunnan, Ministry of Education Key Laboratory of Agricultural Biodiversity for Plant Disease Management, College of Plant Protection, Yunnan Agricultural University, 452 Fengyuan Road, Kunming 650201, China; Institute of Tropical and Subtropical Cash Crops, Yunnan Academy of Agricultural Sciences, 518 Lancheng Road, Baoshan 678000, China; National Key Laboratory of Tropical Crop Breeding, Institute of Tropical Crop Genetic Resources, Chinese Academy of Tropical Agricultural Sciences, 4 Xueyuan Road, Haikou, Hainan 571101, China; Institute of Tropical and Subtropical Cash Crops, Yunnan Academy of Agricultural Sciences, 518 Lancheng Road, Baoshan 678000, China; Institute of Tropical and Subtropical Cash Crops, Yunnan Academy of Agricultural Sciences, 518 Lancheng Road, Baoshan 678000, China; Institute of Tropical and Subtropical Cash Crops, Yunnan Academy of Agricultural Sciences, 518 Lancheng Road, Baoshan 678000, China; Laboratory of Plant Genetic and Cell Biology, Faculty of Plantation and Agrotechnology, Universiti Teknologi MARA, Jasin Campus, 77300 Merlimau, Melaka, Malaysia; State Key Laboratory for Conservation and Utilization of Bio-Resources in Yunnan, Ministry of Education Key Laboratory of Agricultural Biodiversity for Plant Disease Management, College of Plant Protection, Yunnan Agricultural University, 452 Fengyuan Road, Kunming 650201, China; National Key Laboratory of Tropical Crop Breeding, Institute of Tropical Crop Genetic Resources, Chinese Academy of Tropical Agricultural Sciences, 4 Xueyuan Road, Haikou, Hainan 571101, China

##  

Dear Editor

Mango (*Mangifera indica*) is grown in many tropical and subtropical regions worldwide, and is renowned for its rich flavor and nutritional value. Over recent years, several mango genome assemblies have been released, providing foundational information for genomics-based mango breeding [1–4]. However, only genomes for commercial accessions are reported in these studies, and publications are lacking on genome assembly of mango landraces , whose value in breeding has been demonstrated in many other crops [5]. Recent advances in sequencing technology have made it possible to construct gapless genome assemblies, offering insights into genome evolution and gene functioning [6]. As gaps are left in each of the publicly available mango genomes, construction of a gapless assembly would facilitate the utility of mango genomics data for genetics and breeding.

Here we present the gapless genome assembly of a mango landrace, San Nian Mang (hereafter, SNM). Deep sequencing harnessed 31.4 Gb Oxford Nanopore Technologies (ONT) ultra-long reads (80×), 13.7 Gb PacBio Sequel II HiFi reads (40×), 29.3 Gb Illumina short reads (80×), and 81.1 Gb Hi-C sequencing data (200×). Hybrid genome assembly was performed using HiFi reads and ONT ultra-long reads, yielding a draft assembly of 380.8 Mb [7]. The assembly was scaffolded and further corrected, generating 20 pseudomolecules. After filling all the gaps, a gapless genome containing 20 chromosomes with a total length of 377.6 Mb was generated. This genome contains centromeric sequences in all the 20 chromosomes, and telomeric sequences in 37 of the 40 chromosome termini. Telomeres were unidentified only in short arms of the 10th, 11th and 16th chromosomes, all of which are very close to centromeric regions (Fig. 1A). The contiguity, completeness, and accuracy were confirmed by calculating the contig/chromosome ratio, read mapping, and BUSCO (Benchmarking Universal Single-Copy Orthologs) analyses, respectively (Fig. 1C) [8, 9]. A total of 126.7 Mb of repetitive sequences accounts for 33.5% of the whole genome sequence, with long terminal repeats (LTRs) accounting for remarkably larger percentages of repetitive sequences in centromeres (80.3%) than in other regions (54.7%) of the SNM genome (Fig. 1B and C). Also, centromeric regions showed higher proportions of LTRs and lower sequence complexity than other chromosome regions (Fig. 1D). Copia retrotransposons represent the major LTR type in centromeres of all the chromosomes except Chr10, in which Gypsy is the main type. A total of 38 726 protein-coding genes were identified in the SNM genome (Fig. 1C). The density of protein-coding genes in chromosomes is largely negatively correlated with that of repetitive elements (*R*^2^ = 0.59, *P* < 2.2e−16) and positively correlated with GC content (*R*^2^ = 0.04, *P* = 4.99e−4). Collectively, we obtained a gapless genome assembly for a mango landrace, demonstrating superior assembly and annotation quality. To our knowledge, this is the first reported gapless mango genome assembly.

**Figure 1:**
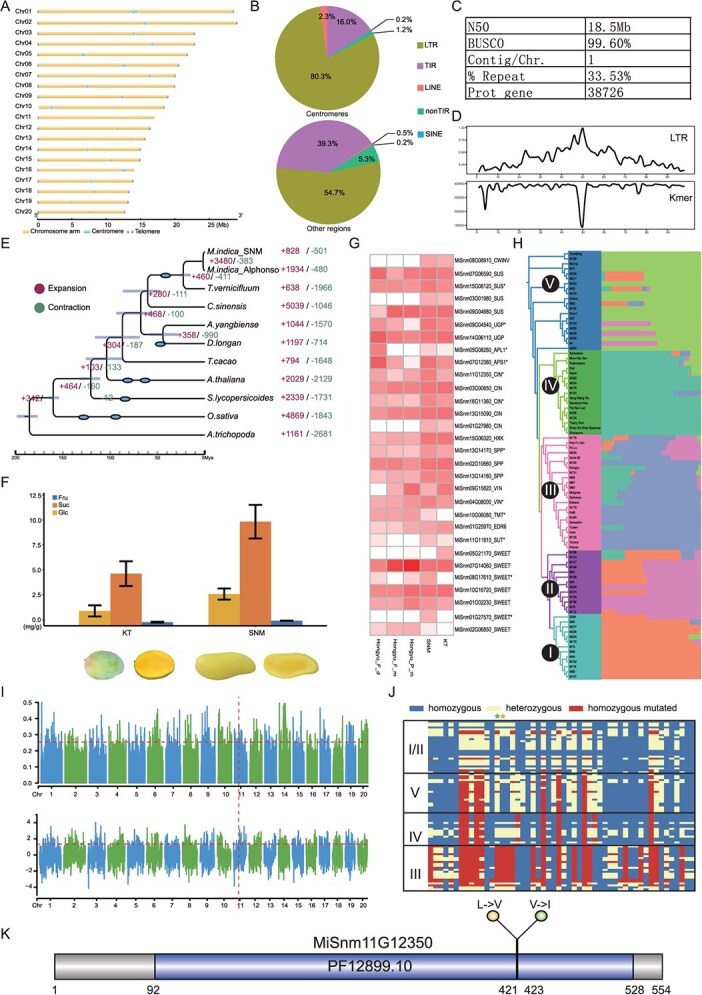
Gapless genome of mango landrace San Nian Mang (SNM) and population genomics analyses. (A) Diagram of the assembled chromosomes of the gapless SNM genome, with centromeric regions and telomeric sequences highlighted. (B) Composition of repetitive elements in centromeres and other genome regions. (C) Statistics of SNM genome assembly and annotation. (D) LTR (above) and *K*-mer (below) distribution in Chr14, showing major peaks in centromeric region. (E) Evolutionary relationships of SNM mango, Alphonso mango, and nine other species with divergence estimated. Numbers of significantly expanded and contracted genes are shown at nodes and tips of the phylogenetic trees, and ovals in the tree represent occurrence of whole-genome duplication events. (F) Contents of sucrose (Suc), glucose (Glc), and fructose (Fru) in Keitt (KT) and SNM fruits. Below are pictures (compact and longitudinal section) of KT and SNM fruits. (G) Expression of carbohydrate biosynthesis genes quantified based on RNA-Seq, with that of a *CIN* gene (MiSnm11G12350) included. From left to right are represented expression levels of Hongyu unripe flesh, Hongyu ripe flesh, Hongyu peels, SNM flesh, and KT flesh. Genes with expression levels significantly higher in SNM flesh than in KT flesh are marked with asterisks. (H) Phylogenetic relationships (left) and population structure (right) of mango germplasms at *K* = 5, both showing distinctiveness of each clade. Labels of clades I–V are equivalent to those in Supplementary Data Figs 13 and 14. SNM is in clade I. (I) Fixation index (*F*_ST_) (upper) and XPEHH (lower) across the SNM genome. The dashed horizontal lines indicate the top 5% thresholds. The dashed vertical line represent signal overlapping with the *CIN* gene. (J) Genotypes of SNPs in selective sweeps within the *CIN* gene sequence. Labels of clades I–V are equivalent to those in Supplementary Data Figs 13 and 14. The SNPs responsible for alterations of amino acid residues are marked with asterisks. (K) Diagram of CIN peptide sequence, showing changes of amino acid residues resulting from nucleotide variation, together with the conserved domain.

Self-synteny, *K*_s_ and 4Dtv analyses all supported occurrence of a recent duplication event in the SNM genome. Co-synteny analysis showed a high collinearity among mango genome assemblies, but there are a number of rearrangements, including inversions and intra-chromosomal translocations. Phylogenetic inferences resolved the two mango varieties and the lacquer tree as a monophyletic group, which represent the family Anacardiaceae (Fig. 1E). The mango split from the lacquer tree at 16.9–27.3 Mya, which is between the end of the Oligocene and the beginning of the Miocene period (Fig. 1E). Comparative genomics showed that 828 gene families were significantly expanded in the SNM genome (Fig. 1E). As a result of functional enrichment analyses, terms related to signaling transduction, epigenetic modification, and metabolite processing were enriched, highlighting the differentiation of genome content, which may affect the cellular physiological system shaping the split of the landrace SNM and the commercial variety Alphonso. These results jointly show the divergence of the mango landrace from other germplasms despite high synteny among mango varieties.

Local consumers praise SNM fruits, which taste particularly sweet compared with many typical mango varieties. We measured sugar contents in ripe fruit flesh of SNM and that of a commercial variety, Keitt, and results showed that soluble sugar contents in SNM fruit flesh were significantly higher than those in Keitt (Fig. 1F). We further quantified gene expression levels in ripe fruit flesh in SNM versus those in Keitt based on transcriptomic analysis, and KEGG enrichment analysis showed that the up-regulated genes were significantly enriched in sugar synthesis. We identified gene homologs encoding enzymes in the soluble sugar biosynthesis and transport pathway, and transcriptomic analysis showed that expression levels of several genes were significantly higher in SNM than in Keitt fruits, a commercial variety with Indian origin (Fig. 1G). These results demonstrate the chemical and gene expression basis of the sweet taste trait of SNM based on our SNM genome annotation.

To further characterize the genomic basis of the significant sugar accumulation, we obtained resequencing data of 90 mango accessions, including 58 accessions generated within this study, and 32 accessions released along with our previous publication, which were used for population structure inference [3]. After applying filtering criteria, a total of 9 029 413 SNPs and 382 676 indels were identified. Based on the SNPs, six distinct monophyletic clades, including a clade representing the *Mangifera* relatives, were resolved with phylogenetic analysis. Rooted with the relatives, the mango germplasms were split into two major groups, of which one contains those of Indian peninsula origin and one harbors those of Southeast Asia origin, supporting previous reports that the mango has two diversity centers. Principal component analysis (PCA), excluding *Mangifera* relatives, clustered the germplasms into five distinct groups, consistent with the phylogenetic topology. When *K* = 5, the best model revealed by cross-validation error analysis, population structure showed the distinct groups largely consistent with the phylogenetic analysis and PCA analysis (Fig. 1H). Population structure analysis also showed that the mango germplasms widely experienced allelic admixture. For example, Mulgoba, Alphonso, and Edward, which were grouped with the germplasms of South Asia origin, showed admixture with germplasm of Southeast Asia origin (Fig. 1H). SNM, a germplasm collected along Nujiang River for deep sequencing in this study, is grouped with other germplasms collected near Nujiang River. However, population structure analysis suggested introgression from germplasms near Lancang River to SNM (Fig. 1H). The resequencing-based analyses reveal complex population genetics of mango germplasms with Southeast Asia origin, including the landrace germplasms.

Based on the results of analytic chemistry, gene expression, and population genetics, we further sought to identify signals and genes in the SNM genome that are potentially related to high sugar content in SNM fruits. We mined genes experiencing genetic sweep, among which 11 genes associated with sugar biosynthesis overlapped with genomic regions of genetic sweep by *F*_ST_ and cross-population extended haplotype homozygosity (XPEHH) analyses, respectively. A gene (MiSnm11G12350) encoding cell wall invertase (CIN) is located in a genetic sweep region in Chr11 (Fig. 1I). CIN has been well characterized as a key enzyme in sucrose metabolism in other species [10]. Within this gene, we found multiple SNPs showing evident differences of single nucleotides between germplasm of South and Southeast Asia origins (Fig. 1J). Two nucleotide variations are located in the conserved domain (PF12899.10) of the peptide sequence of the gene, both of which are non-synonymous mutations leading to changes of amino acid residues (Fig. 1K). Interestingly, this gene was expressed significantly more highly in SNM fruits than in Keitt ones (Fig. 1G). The genetic sweeps and key genes identified here provide a basis for the genetic improvement of mango for the alteration of fruit sugar content, a key trait of mango as a fruit crop.

In conclusion, we generated a gapless genome assembly for SNM, a mango landrace. We also analyzed genome resequencing data for local landraces together with those of commercial varieties, and population genomics analyses resolved several significant groups, suggesting multiple domestication events for mango. Resequencing analyses also suggest considerable genomic variations, and variants in a gene encoding cell wall invertase, a key enzyme in sugar accumulation in mango fruits, were discovered. Our results highlight the significance of mango landraces, which harbor valuable genetic diversity that can be harnessed for mango improvement and sustainable agriculture.

## Data Availability

Raw data, genome assembly and annotation data are available under National Center for Biotechnology Information studies ID PRJNA1112139 (HiFi, ONT, HiC, RNASeq, Illumina sequencing data, genome assembly and annotation) and PRJNA1117965 (genome resequencing).
